# Stem cells of ependymoma

**DOI:** 10.1038/sj.bjc.6603519

**Published:** 2006-12-19

**Authors:** H Poppleton, R J Gilbertson

**Affiliations:** 1 Department of Developmental Neurobiology, St Jude Children's Research Hospital, 332 N Lauderdale Street, Memphis, TN 38105, USA; 2 Department of Oncology, St Jude Children's Research Hospital, 332 N Lauderdale Street, Memphis, TN 38105, USA

**Keywords:** stem cell, ependymoma, brain, tumour, CD133 radial glia

## Abstract

Ependymomas are tumours that arise throughout the central nervous system. Little is known regarding the aberrant cellular and molecular processes that generate these tumours. This lack of knowledge has hampered efforts to reduce the significant mortality and morbidity that are associated with ependymoma. Here, we review recent data that suggest that radial glia are cells of origin of ependymoma, and discuss the processes that might transform these neural progenitors into ependymoma cancer stem cells.

Ependymomas are central nervous system (CNS) tumours that originate from the wall of the ventricular system along the entire craniospinal axis (Kleihues *et al*, 2002). Although ependymomas from different regions of the CNS are histologically similar, they are clinically (Moynihan, 2003) and genetically (Ebert *et al*, 1999; Dyer *et al*, 2002; Taylor *et al*, 2005) distinct, suggesting that they represent a collection of different diseases. Intracranial ependymomas often recur at the primary tumour site and less than 60% of children with this disease will survive more than 5 years (Gatta *et al*, 2002). Despite this significant clinical burden, we have learned almost nothing new about the biology and treatment of ependymoma during the last 20 years. Indeed, less than 5% of the 14 200 brain tumour research studies published in the last 5 years have investigated ependymoma. Unless we can increase understanding of the biology of ependymoma we are unlikely to reduce the morbidity and mortality associated with this disease.

## CANCER STEM CELLS

Cancer researchers have invested a great deal of effort in characterising the genetic alterations that accumulate in end-stage tumours. Although these studies can provide lists of the genetic events that contribute to the expansion of malignant clones, they do not inform us regarding the chronology or relative importance of each of these alterations in the cancer process. A more comprehensive understanding of cancer that spans the life of the disease from the birth of the first malignant cell to clinical presentation, would be invaluable in the hunt for more effective treatments of cancer. With this in mind, considerable excitement has surrounded the recent discovery of cancer stem cells (CSC) (Clarke and Fuller, 2006). Cancer stem cell make up just a small fraction of the total population of the malignant cells in many solid tumours and leukaemias. However, evidence indicates that these self-renewing and multipotent stem cell-like cells generate all of the phenotypically diverse cells that populate tumours (Lapidot *et al*, 1994; Bonnet and Dick, 1997; Al-Hajj *et al*, 2003; Singh *et al*, 2004; Taylor *et al*, 2005). The discovery of CSC has therefore provided researchers with a practical point of focus for studying the natal cellular and molecular events of tumorigenesis.

The identification of CSC is likely to have important implications for the treatment of cancer. If tumours are derived entirely from CSC, then it would follow that to be curative, cancer treatments should disable or destroy these cells. Indeed, drugs that are designed to kill CSC could prove highly effective treatments of cancer. On the other hand, evidence that CSCs are remarkably similar to normal stem cells predict that such treatments may also possess significant toxicities. For example, brain CSC express the neural stem cell markers Nestin and CD133 (Hemmati *et al*, 2003; Galli *et al*, 2004; Singh *et al*, 2004; Taylor *et al*, 2005), whereas acute myeloid leukaemic CSC display the CD34^+^/CD38^−^ immunophenotype of haematopoietic stem cells (HSC) (Lapidot *et al*, 1994; Bonnet and Dick, 1997). Thus, the development of safe and effective therapies for all cancers is likely to require understanding of the similarities and differences between normal and malignant stem cells in tissues.

## RADIAL GLIA ARE CANDIDATE CELLS OF ORIGIN OF EPENDYMOMA CSC

Comparative studies of normal and malignant HSC have been facilitated by the availability of robust assays for all stages of haematopoiesis (Warner *et al*, 2004). In contrast, knowledge of the stem and progenitor cell populations of non-haematologic organs is limited. Therefore, identifying the cell of origin of solid tumour CSC and the molecular alterations that transform these cells is less straight forward. Recently, we compared the gene expression profiles of developing tissues and ependymoma subsets to identify populations of cells in the CNS which act as the cells of origin of ependymomas (Taylor *et al*, 2005).

In an analysis of over 100 ependymomas, we found that tumour subsets exhibit distinct patterns of gene expression and regions of chromosome gain and loss that correlate with the anatomic location of the tumour (supratentorial region, posterior fossa or spine) (Taylor *et al*, 2005). Gene expression signatures that most discriminated supratentorial, posterior fossa and spinal ependymoma included many genes that are known regulators of neural precursor cells in the corresponding region of the CNS. For example, we found that supratentorial tumours express markedly elevated levels of members of the EPHB-EPHRIN and NOTCH cell signal systems that play key roles in maintaining normal neural stem cells in the cerebral subventricular zone (SVZ) (Conover *et al*, 2000; Hitoshi *et al*, 2002). Conversely, spinal ependymomas expressed multiple Homeobox (*HOX*) family members that coordinate antero-posterior tissue patterning and development of the spine (Dasen *et al*, 2003; Kmita and Duboule, 2003). Therefore, we reasoned that subsets of ependymoma either maintain, or recapitulate the developmental expression profiles of anatomically restricted progenitor cells. To identify these precursor cells in the normal CNS, we used *in situ* hybridisation and immunofluorescence to map the site of expression of ependymoma signature genes in the developing mouse. These data identified remarkable similarities between the distinct gene expression patterns observed in embryonic radial glia (RG) – that are neural progenitor cells (see below) – and those observed in human ependymomas from the corresponding region of the CNS. The great majority of intracranial tumours in our study arose in children; whereas the spinal tumours were obtained from adult patients. Therefore, our data suggest that RG are a likely source of ependymomas independent of patient age. It is noteworthy that astroglial cells with functional and molecular characteristics of RG persist in the SVZ of the lateral ventricles and possibly the spinal cord, suggesting that some RG give rise to adult neural stem cells (Merkle *et al*, 2004; Barry and McDermott, 2005). It is possible that these RG-derived stem cells are cells of origin of adult ependymomas. We are conducting extensive genomic and functional studies of stem cell populations in normal and malignant embryonic and adult neural tissues to identify the precise cell of origin of each type of ependymoma.

Importantly, we showed also that self-renewing and multipotent CSC isolated from fresh samples of ependymoma are: bipolar RG-like cells; express the CD133+/Nestin+/RC2+/brain lipid-binding protein (BLBP)+ immunophenotype of RG; and are both required and sufficient to generate tumours *in vivo*. Our data suggest a new hypothesis for the origin of ependymoma: that RG in different parts of the CNS are predisposed to acquire distinct genetic abnormalities that transform these cells into CSC of supratentorial, posterior fossa and spinal ependymoma.

## WHAT ABERRANT PROCESSES TURN RG INTO CSC?

To determine whether ependymoma CSC arise from RG, and how this malignant transformation may occur, it is important to understand the processes that regulate the generation and fate of RG in the CNS. In vertebrates, neural stem cells first appear as a layer of pseudostratified epithelium that lines the neural plate and neural tube before the onset of neurogenesis. These neuroepithelial cells (NEC) are highly polarised along their apical–basal axis (Gotz and Huttner, 2005). Of particular note, the apical cell membrane of NEC contains characteristic transmembrane proteins, for example, prominin-1 (the mouse orthologue of human CD133), and adjacent adherens junctions that are thought to regulate cell proliferation and fate decisions.

As neurogenesis begins, NEC give rise to RG. RG retain the highly polarised features of NEC and express CD133, Nestin and RC2, but they also express astrocyte-specific proteins that include glutamate transporter and BLBP (Kriegstein and Gotz, 2003). Previously, RG were believed to generate only astroglial cells. However, studies conducted within the last 5 years indicate that RG are mitotically active, multipotent progenitor cells that probably give rise to the majority of neurons, astrocytes, oligodendrocytes and ependymal cells in the brain (Fishell and Kriegstein, 2003; Gotz and Huttner, 2005; Spassky *et al*, 2005).

**Figure 1:**
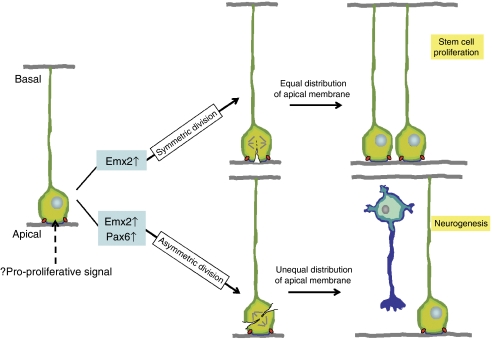
Symmetric and asymmetric division of NEC and RG. Neuroepithelial cell and RG (green) are highly polarised. The apical membrane (blue) is flanked by adherens junctions (red ovals) that are thought to transduce pro-proliferative signals and determine daughter cell fate. *EMX2* promotes symmetric division (in embryonic stem cells) that distributes the apical membrane equally to the two daughter cells, resulting in stem cell proliferation. Both Emx2 (in adult neural stem cells) and Pax6 can promote asymmetric division. Only the daughter cell that inherits the apical membrane resembles the mother cell, the remaining daughter is fated to differentiate (blue, e.g. neuron).

Neuroepithelial cells and RG each possess the capacity for symmetric and asymmetric cell division (Figure 1). Symmetric division expands the neural stem and progenitor cell pool, as this generates two identical daughter cells that resemble the parent NEC or RG cell. In contrast, asymmetric divisions generate unequal daughter cells: one stem cell and one cell that is fated to differentiate. In normal tissues, stem cell self-renewal – cycles of division that repeatedly generate at least one daughter equivalent to the mother cell – is tightly regulated, and deregulation of this process is emerging as a key event in the development of CSC (Clarke and Fuller, 2006). Thus, if ependymoma CSC arise from RG, then the factors that coordinate RG cell division might represent targets for mutations that underlie the initiation of this brain tumour.

### Aberrant RG cleavage

**Figure 2:**
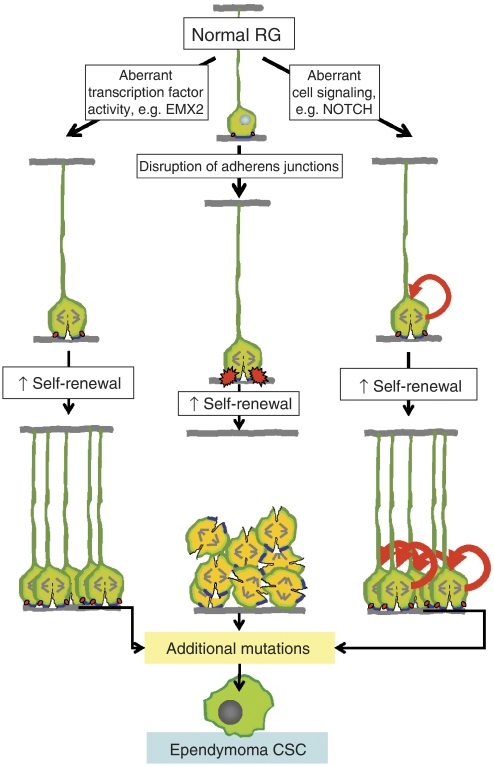
Potential mechanisms of RG transformation. Aberrant RG cleavage mediated by certain transcription factors, disruption of adherens junctions and deregulation of pro-proliferative cell signals could each accelerate RG self-renewal and expansion of the stem cell pool. In cooperation with additional mutations these events might result in the generation of CSC.

The apical plasma membrane appears to play an important role in determining the fate of NEC and RG daughter cells (Gotz and Huttner, 2005). Daughter cells that inherit the apical membrane retain the proliferative stem cell properties of the parent cell (Figure 1). Thus, it has been suggested that the apical membrane acts as a transducer of pro-proliferative signals from the neural tube to NEC and RG (Gotz and Huttner, 2005). Indeed, equal distribution of the apical membrane between the progeny of symmetrically dividing RG, might explain why this division results in identical daughter cells with proliferative progenitor cell properties. Whether RG undergo symmetric or asymmetric division appears to be controlled, at least in part, by certain transcription factors (Figure 1). For example, *Emx2* induces symmetric divisions that can lead to expansion of RG cell numbers, whereas *Pax6* activates neurogenic, asymmetric division (Heins *et al*, 2001, 2002). Interestingly, we found that expression of *EMX2*, but not *PAX6*, is markedly upregulated in supratentorial ependymomas compared to ependymomas from other regions of the CNS (37-fold expression difference *P*<0.0001; Taylor *et al*, 2005). Thus, aberrant and prolonged expression of *EMX2* within embyonic cortical RG might contribute to increased symmetric cell division and the formation of CSC of pediatric supratentorial ependymoma (Figure 2). One important caveat against these rather simple models of tumourigenesis, is the capacity of genes to confer context dependent effects on stem cells. For example, studies have shown that *Emx2* can operate as a negative regulator of symmetric cell division in adult neural stem cells (Galli *et al*, 2002). Better understanding of the precise cell of origin of ependymomas should help to clarify the role of specific genes in the development of these diseases.

### Disruption of RG adherens junctions

Neuroepithelial cell and RG each contain concentrations of adherens junctions that are located immediately beneath the apical plasma membrane (Gotz and Huttner, 2005). Evidence indicates that disruption of these complexes could also contribute to the transformation of RG (Figure 2). Indeed, deletion of the essential adherens gene *αE-Catenin* from neural progenitor cells in mice results in severe disruption of the apical cell junctions, loss of cell polarity, increased stem cell proliferation and the formation of tumour-like masses in the brain (Lien *et al*, 2006). It remains to be determined whether disruption of adherens complexes, that occurs frequently in human cancers, might contribute to the formation of CSC in the brain (Cavallaro and Christofori, 2004).

### Deregulation of cell signal pathways

Certain cell signal pathways that control RG self-renewal are deregulated in ependymoma. Most notable among these is the NOTCH cell signal pathway. Upregulation of the Notch ligand Jagged1 maintains the self-renewal and multipotency of adult neural stem cells, whereas deletion of Notch1 depletes the neural stem cell fraction (Hitoshi *et al*, 2002; Nyfeler *et al*, 2005). Further, retroviral transfer of activated Notch1 into cells lining the SVZ of the forebrain of embryonic mice promotes the formation and maintenance of RG (Gaiano *et al*, 2000). Interestingly, the Notch cell signal pathway might serve as a link between the symmetry of NEC and RG division and the fate of daughter cells. In this regard, deletion of *Lethal Giant Lavae-1* from dividing neural stem cells of mice, prevents the asymmetric localisation the Notch inhibitor Numb to daughter cells, resulting in failure of asymmetric cell divisions and the formation of tumour-like masses within the brain (Klezovitch *et al*, 2004). We found that the signature genes that are most upregulated in supratentorial ependymomas include the NOTCH ligands JAGGED 1 and 2, and the NOTCH signal targets HES1 and HES5 (Taylor *et al*, 2005). Interestingly, the *ERBB2* oncogene that is expressed to high levels in ependymoma, is an important target of Notch signalling in RG (Gilbertson *et al*, 2002; Ever and Gaiano, 2005). Downregulation of ErbB2 expression or activity in RG causes these cells to differentiate, whereas activation of ErbB2 maintains RG proliferation. Thus, deregulation of NOTCH signalling, either alone, or in concert with other cell signal molecules might promote the formation of ependymoma CSC (Figure 2).

p19^Arf^ and p16^Ink4a^ that are encoded by the *Ink4a/Arf* locus, are two additional regulators of neural stem cell proliferation. In this regard, Bmi1 promotes the self-renewal of neural stem cells by repressing transcription at the *Ink4a/arf* locus; whereas deletion of *Ink4a* significantly expands the neural progenitor cell population (Bruggeman *et al*, 2005; Molofsky *et al*, 2005). We found that concurrent activation of NOTCH cell signalling and deletion of *INK4A/ARF* affect the great majority of supratentorial ependymomas. Specifically, using array comparative genomic hybridisation and fluorescence *in situ* hybridisation, we have shown that *INK4A/ARF* is selectively deleted from >90% of tumour cell nuclei of supratentorial ependymomas but is rarely deleted from tumours arising in other regions of the CNS (Taylor *et al*, 2005). Thus, cortical RG might be susceptible to transformation into ependymoma CSC by concurrent activation of NOTCH signalling and deletion of *INK4A/ARF*.

Further studies will be required to characterise fully the factors that contribute to the formation of ependymoma CSC. These efforts are likely to be assisted by ongoing studies of flies that have identified already tumour suppressor genes that regulate the proliferation of neural stem cells. For example, the *Drosophila* tumour suppressor protein Brat is normally distributed asymmetrically to one of the daughters of dividing neural stem cells, fating that cell to differentiate, whereas the remaining daughter cell self-renews (Betschinger *et al*, 2006). In *Drosophila brat* mutants both daughter cells self-renew, resulting in expansion of the stem cell pool and the formation of larval brain tumours.

## EPENDYMOMA CSC AND THE CLINIC

If the CSC hypothesis proves correct and brain tumours, including ependymoma, arise from rare fractions of stem-like cancer cells, then these findings will lead to a paradigm shift in the way we treat CNS tumours. In particular, we should begin to develop classification systems and targeted treatment strategies that focus on the eradication of CSC. This strategy may prove particularly effective in tumours such as ependymoma that include developmentally and molecularly distinct subgroups that are unlikely to respond uniformly to all treatments.

At least two approaches might be adopted in the development of anti-CSC therapies. First, as normal stem cells are protected from environmental insults by both cell intrinsic and extrinsic factors, then CSC might be inherently resistant to conventional chemo- and radiotherapies (Dick and Lapidot, 2005). Thus, agents that counteract CSC drug resistance mechanisms might prove useful in the treatment of cancer. Second, targeting the pathways that regulate aberrant self-renewal could be used to disable or destroy CSC. Clinical trials of one such class of drugs, inhibitors of *γ*-secretase, are currently underway among patients with leukaemia, and plans to trial these agents among children with ependymoma are in advanced stages within the US Pediatric Brain Tumor Consortium. NOTCH signalling is activated following *γ*-secretase mediated cleavage of the NOTCH receptor (Radtke and Raj, 2003). Thus, inhibitors of *γ*-secretase might be effective treatments of supratentorial ependymomas.

The success of anti-CSC therapies will require not only that these drugs kill or disable CSC, but also that they spare normal stem cells. This issue is especially important when considering the treatment of children with brain tumours whose nervous system is still developing. Thus understanding further the origins of CSC in the brain and how best to target these in the clinic will likely require the ongoing collaboration of developmental biologists, cancer biologists and clinicians.
